# All-arthroscopic versus mini-open repair of small or moderate-sized rotator cuff tears: A protocol for a randomized trial [NCT00128076]

**DOI:** 10.1186/1471-2474-7-25

**Published:** 2006-03-10

**Authors:** Joy C MacDermid, Richard Holtby, Helen Razmjou, Dianne Bryant, JOINTS Canada

**Affiliations:** 1 School of Rehabilitation Science, McMaster University, 1400 Main St. West, IAHS-403, Hamilton, Ontario, L8S 1C7, Canada; 2 Hand and Upper Limb Centre Clinical Research Laboratory, St. Joseph's Health Centre, 268 Grosvenor St., London, Ontario, N6A 3A8, Canada; 3 Orthopaedic & Arthritic Hospital, Sunnybrook & Women's College Health Sciences Centre, 43 Wellesley St. East, Toronto, Ontario, M4Y 1H1, Canada; 4 Department of Physical Therapy, Elborn College, University of Western Ontario, London, ON N6G 1H1, Canada; 5 Joint Orthopaedic Initiative for National Trials of the Shoulder – Canada

## Abstract

**Background:**

Rotator cuff tears are the most common source of shoulder pain and disability. Only poor quality studies have compared mini-open to arthroscopic repair, leaving surgeons with inadequate evidence to support optimal, minimally-invasive repair.

**Methods/Design:**

This randomized, multi-centre, national trial will determine whether an arthroscopic or mini-open repair provides better quality of life for patients with small or moderate-sized rotator cuff tears. A national consensus meeting of investigators in the Joint Orthopaedic Initiative for National Trials of the Shoulder (JOINTS Canada) identified this question as the top priority for shoulder surgeons across Canada. The primary outcome measure is a valid quality-of-life scale (Western Ontario Rotator Cuff (WORC)) that addresses 5 domains of health affected by rotator cuff disease. Secondary outcomes will assess rotator cuff functionality (ROM, strength, Constant score), secondary dimensions of health (general health status (SF-12) and work limitations), and repair integrity (MRI). Outcomes are measured at baseline, at 6 weeks, 3, 6, 12, and 24 months post-operatively by blinded research assistants and musculoskeletal radiologists. Patients (n = 250) with small or medium-sized cuff tears identified by clinical examination and MRI who meet eligibility criteria will be recruited. This sample size will provide 80% power to statistically detect  a clinically important difference of 20% in WORC scores between procedures after controlling for baseline WORC score (α = 0.05). A central methods centre will manage randomization, data management, and monitoring under supervision of experienced epidemiologists. Surgeons will participate in either conventional or expertise-based designs according to defined criteria to avoid biases from differential surgeon expertise. Mini-open or all-arthroscopic repair procedures will be performed according to a standardized protocol. Central Adjudication (of cases), Trial Oversight and Safety Committees will monitor trial conduct. We will use an analysis of covariance (ANCOVA), where the baseline WORC score is used as a covariate, to compare the quality of life (WORC score) at 2 years post-operatively. As a secondary analysis, we will conduct the same statistical test but will include age and tear size as covariates with the baseline score. Enrollment will require 2 years and follow-up an additional 2 years. The trial will commence when funding is in place.

**Discussion:**

These results will have immediate impact on the practice behaviors of practicing surgeons and surgical trainees at JOINTS centres across Canada. JOINTS Canada is actively engaged in knowledge exchange and will publish and present findings internationally to facilitate wider application. This trial will establish definitive evidence on this question at an international level.

## Background

### Rotator cuff is essential to function, but at risk of injury

The shoulder joint is essential for placing the hand in functional positions and as such requires mobility, strength, and stability. To provide mobility, the joint has a small shallow glenoid upon which the humeral head is permitted a large range of movement. Unlike other socket joints like the hip where bony structure provides stability, in the shoulder, stability is provided primarily by muscles. This defines the critical importance of the rotator cuff muscles and the cascade of events that follow when cuff integrity is compromised. The rotator cuff muscles are responsible for dynamic stabilization of the glenohumeral joint during shoulder motion. During abduction, the supraspinatus and the deltoid muscle work synergistically to elevate the arm. The infraspinatus, subscapularis, and teres minor depress the humeral head and maintain joint stability by opposing the upward forces created by the supraspinatus and deltoid. A cuff tear can occur after a single extreme overload, but typically it is the result of a degenerative process that compromises tendon integrity, starting with supraspinatus [1] and progressing to the other cuff muscles. A rotator cuff tear destroys the balance of forces at the shoulder and as a result, the humeral head is driven superiorly, resulting in pain, loss of strength, and reduced motion – all of which contribute to a profound functional loss. The progression of a complete cuff tear leads to rotator cuff arthropathy (osteoarthritis).

### There is a high incidence of tears

It has been established that degeneration, i.e., frictional wear in the presence of hypovascularity, is the primary mechanism of rotator cuff tears [1-4] with anatomic factors, i.e., acromial shape, having a smaller or uncertain role [5,6]. Thus, aging and overuse both contribute to high rates of cuff pathology. There is consensus across epidemiological studies that full thickness tears increase with age [1-3,7-9], with some studies reporting an incidence of 5% in patients in their fourth decade and 80% of those in their eighth decade [1-3,5,7,10]. While rates vary across studies, there is consensus that incidence is high and that changes are often bilateral and age dependent.

### A cuff tear has a large effect on disability and quality of life

The impact of rotator cuff pathology on overall health, work productivity, and quality of life is profound. A study of over 500 shoulder problems, including 111 rotator cuff tears, indicated that health status was equally compromised in these patients as compared to 5 major medical diagnoses (hypertension, congestive heart failure, acute myocardial infarction, diabetes mellitus, and clinical depression) [11]. Others have confirmed the impact of cuff disease on health at the primary care level [12]. A substantial loss in work productivity is evident in the high claims rates and costs attributed to cuff disease in the worker's compensation system [9,13,14], where cuff pathology ranks second to back pain as a reason for lost time from work in manual workers [14]. Cuff pathology results in high levels of pain, disability, and reduced quality of life compared to age-matched controls [15]. It has been shown that the presence of cuff pathology is a primary determinant of health status (SF-36) (R^2 ^= 72%) [15]. Furthermore, rotator cuff surgery has been shown to have a significant impact; not just on shoulder symptoms, but also on overall health status [16].

### Studies on arthroscopic and mini-open repairs are low quality and inconclusive

There has been a move across surgical disciplines to minimize the extent of surgical dissection required to perform procedures. Procedures that have traditionally been performed though open techniques are now performed arthroscopically or through mini-open procedures. For example, there have been sufficient numbers of randomized controlled trials (RCTs) comparing open and endoscopic carpal tunnel release that systematic reviews have now been completed on this topic [17]. Recently, both arthroscopic and mini-open procedures have been introduced into shoulder surgery. While both interventions are less invasive than open procedures, there are variable advantages and concerns with these two procedures [18]. Repair by an all-arthroscopic procedure is less invasive, but requires more extensive training [18]. While a more rapid recovery might be expected, the adequacy of the repair and risk of complications have been questioned [19]. Some surgeons are reluctant to invest time in training for this procedure, unless it can be shown that an all-arthroscopic repair provides superior results [18,20].

A few low-quality (level 4) case series have evaluated the outcomes of these two surgical interventions. A completely arthroscopic technique has been reported as effective across a spectrum of tears [21-28], specifically in small to medium [21], moderate to large [29], and large/massive tears [19]. There is consensus across these studies that clinical improvements can be obtained in the majority of cases, although a recurrent defect was reported in 17/18 large or massive tears [19]. Similarly, other cases series report a high percentage of good/excellent results in patients treated with arthroscopically assisted mini-open repairs across a spectrum of tear sizes, specifically for both small/moderate to large [30] tears.

Three low-quality (level 3) studies have reported head-to-head comparison of mini-open versus all-arthroscopic rotator cuff repair [31-33]. Two studies (n = 19 [32] and n = 76 [33]) found similar disability and strength scores at follow-up; another study [31] reported similar long-term disability, but faster return of motion in the arthroscopic group as compared to the mini-open procedure. In these studies [31-33], patients were not randomized, follow-up was retrospective, and in one study [33], patients received a mini-open procedure following "technical failure" of arthroscopic repair. Thus, these low-quality studies from different subpopulations may be biased since groups potentially differ in their prognostic balance. Further, comparison across previous studies is difficult due to variation in patient selection techniques, symptom duration, disability levels, and extent of pathology. To date, no randomized clinical trial has attempted to compare the results of an all-arthroscopic repair to an arthroscopically assisted mini-open repair. High-quality evidence is required to assist surgeons to determine whether the move towards less invasive procedures in cuff repair is appropriate.

### There are challenges in conducting surgical trials in orthopaedic surgery

There are a number of barriers to the conduct of randomized clinical trials in surgical practices. One such barrier is the influence of surgeon skill as a component of the treatment process [34]. Surgeons are rarely equally competent at two different surgical procedures for the same clinical problem, and even more rarely do they believe that two surgical options are equally beneficial [34]. Thus, the classic randomized trial developed in medicine to evaluate drug therapies can fall short when applied to the evaluation of surgical interventions, where skill and preference for a particular procedure have the potential to bias the results or challenge the feasibility of the trial. In fact, it has been shown that surgeons are less likely to participate in Randomized Controlled Trials (RCTs) as compared to other medical specialties [35]. Surgeons may be reluctant to participate in trials because they do not consider themselves to be in a position of equipoise or may be reluctant to enroll all patients because of treatment preferences. High-quality trials require a broad level of participation to achieve the sample sizes required for adequate power.

To address these challenges, shoulder surgeons across Canada formed a national collaborative group focused on multi-centered clinical trials for shoulder conditions (Joint Orthopaedic Initiative for National Trials of the Shoulder) (JOINTS Canada). Most recently, JOINTS Canada obtained CIHR funding to complete strategic planning on the priority research question facing shoulder surgeons and to identify trial methods suited to meet the challenges inherent in surgical trials.

### Expertise-based random allocation may enhance feasibility and validity

In 1980, randomizing subjects to clinicians was first suggested by Van der Linden [36,37]. Since that time, however, this design has been little used. The expertise-based randomized controlled trial has been recently revisited as a potential alternative to minimizing these barriers [34]. In a conventional RCT, both interventions are offered by each health care provider. This works well in drug trials where there is no interaction between the drug and its provider and where the provider can be blinded to the intervention they are dispensing. However, in surgical trials there are problems with this approach that have been recently described by the Expertise-Based Group [34]. The primary concern is that the conventional RCT may actually introduce a bias, due to "differential expertise" within a treatment group that may act as a confounder leading to spurious or biased results. It has been well established that the patients of surgeons who have more experience can expect better outcomes following a surgical intervention. Thus, the patients of surgeons participating in a conventional clinical trial will tend to have better outcomes if they are assigned to the treatment in which their surgeon is more experienced. Greater imbalance in expertise between surgeons compounded by an imbalance in recruitment rates between surgeons will result in greater differential expertise bias and lower trial validity. In fact, extensive experience may be required to achieve superior outcomes. Thus, conducting a specific number of "run-in cases" prior to enrolling patients (used in conventional RCTs) does little to impact on this form of bias.

Not only do surgeons have differential expertise, but also this is often associated with strong beliefs about which intervention is more effective. In fact, initial beliefs of trial surgeons about which intervention is superior has been shown to persist at the end of the trial [34]. Therefore, it is possible that surgeons will introduce differential treatment bias by managing cases with their preferred intervention in a more rigorous manner. Although self-report measures can obviate the impact of surgeon bias on measurement of outcomes, these cannot control for differential treatment bias. Another form of bias tends to manifest itself in the conventional design because a greater number of procedural crossovers (surgeon does not comply with the random assignment) occur when the patient is assigned to the less preferred treatment arm. Increased rates of crossing over also compromise the validity of the RCT results. Finally, from an ethical viewpoint, it is preferable that patients receive care from a surgeon using a procedure that they were experienced in performing.

Despite these potential advantages, the expertise-based design has rarely been used except in cases where the interventions arise from different disciplines [34]. After consulting with experts [34], we devised a strategy to maximize valid participation of trial surgeons. Given the potential for differential expertise bias, we concluded that only surgeons with long-term (>2 years) and recent experience in both procedures were appropriate for the conventional RCT in our study. Those who demonstrated differential expertise or preference are required to participate in the expertise-based RCT. While this requires two designs, it ensures that the potential bias due to differential expertise or preference for one procedure over another is reduced.

### Systematic reviews confirm that treatment is based on inadequate evidence

Two separate research teams have attempted to conduct systematic reviews to summarize the available evidence for management of shoulder problems, including rotator cuff disease. A 1998 systematic review included all randomized trials that addressed shoulder pain and any related treatment intervention and found insufficient evidence to make any specific recommendations with regards to management of rotator cuff disease [38]. A recent systematic review of surgical or conservative interventions included a broader range of evidence (including level 2 and 3 studies) and specifically focused on patients with rotator cuff disease. This review found weak evidence to suggest that open and primary surgery were more effective than arthroscopic debridement and revision surgery [39]. However, these conclusions do little to provide guidance on current practice trends as these procedures have different indications and recent techniques were not addressed. No randomized clinical trials have compared mini-open to an all-arthroscopic repair technique.

### A trial is needed now

The wait-list for this procedure ranges from 6 months to 2 years in Canada, indicating a high demand for effective rotator cuff repair procedures. Canadian shoulder surgeons who are active members of JOINTS Canada obtained funding from CIHR to mount a team-planning meeting where we identified and reached a consensus that this research question was the top priority for shoulder surgeons and their patients. The timing of this clinical trial is critical. An RCT mounted too early will have difficulty recruiting a sufficient number of surgeons with the experience required to perform the study interventions. If the trial is delayed until less invasive surgical techniques become the standard of practice, a large group of patients will be exposed to suboptimal care should this practice prove less effective. Further, it will be more difficult to change clinical practice after this shift has occurred. Currently, surgeons are making decisions about abandoning traditional repairs in the search of the ideal minimally invasive procedure. Without the benefit of high-quality evidence to support these decisions, premature selection may expose patients to potentially useless or risky procedures. Certainly, haphazard incorporation of new surgical approaches exposes the health care system to inefficient allocation of its limited resources.

## Methods/Design

### Primary objective of the trial

To compare the effectiveness of all-arthroscopic to mini-open rotator cuff repair to improve the quality of life of patients with a small or medium-sized rotator cuff tear.

### Secondary objectives

While maximizing the patient's quality of life is clearly the primary objective, the direct intent of repairing the defect in the cuff is to restore its integrity and functionality. Previous work has shown that self-report measures are poor indicators of this functionality when measured by strength [40], motion [40], or imaging [19]. Therefore, these are important secondary outcomes. Generic health issues and specific work limitations are important outcomes that are not fully addressed by the WORC. Therefore, our secondary objectives are to compare the impact of all arthroscopic to mini-open rotator cuff repair on work limitations (WL-26) [41]; general health (SF-12) [42]; physical impairments (range of motion and strength) [15,43,44]; cuff integrity (MRI/ultrasound) [45,46]; and surgical complications.

### Tertiary objective

While both the expertise-based and conventional RCT designs are accepted methodologies and will be implemented in this study to minimize potential biases, it is appropriate to evaluate the impact of these designs on the results of the study. Therefore, we will determine whether the design [34] affects: patients' willingness to participate (or enrollment rates), dropout rates, satisfaction with study participation, and protocol violations (including crossovers).

#### Planned trial interventions

This trial compares two different methods of repairing a torn rotator cuff. Patients will undergo either 1) a mini-open repair where the repair is performed though a small incision and the arthroscope can be used to address problems within the joint (as per traditional diagnostic procedures); or 2) an all-arthroscopic procedure where joint techniques and repair are both performed entirely thought the arthroscope. While many surgeons have used arthroscopy for diagnosis and debridement, repair through the arthroscope is substantially different and requires additional training and experience. Thus, these methods are quite distinct to surgeons. JOINTS Canada surgeons have discussed elements of the repair that can be standardized and those that must be left to surgeon preference. A protocol for surgery/rehabilitation has been established for study centres.

a. *Arthroscopic repair *will consist of subacromial decompression, repair of the torn cuff tendon, and debridement of partial (up to 50%) tears of biceps. If an anatomic repair of the cuff is possible, the area between the articular margin and the greater tuberosity will be decorticated to prepare a bleeding surface for reattachment of the tendon. The anchor selection will be based on the surgeon's personal preference. The number of anchors used will be recorded. After completion of suture anchor placement, braided sutures will be passed through the torn tendon as either mattress or simple sutures at the surgeon's discretion. Side-to-side sutures at the apex of the tear will be used as indicated. The type and number of sutures and suture anchors will be recorded

b. *Mini-open Repair *will follow arthroscopic examination of the glenohumeral joint and subacromial decompression. The arthroscope will be removed and the standard mini-open repair will be performed. A transverse or vertical skin incision at the lateral border of the acromion is made. Exposure of the cuff will be achieved by dissection of the deltoid fascia, and split of the deltoid from the level of the acromion distally for 4 cm. The edges of the tear are resected. The bone between the articular margin and the greater tuberosity is decorticated or a bone trough prepared at the surgeon's discretion. Suture anchors or transosseus sutures may be used along with side-to-side sutures. Details of technique will be recorded as above.

### Subjects

#### Inclusion/Exclusion criteria

We will recruit patients with small or medium-sized rotator cuff tears as determined by clinical examination and diagnostic imaging (MRI) prior to surgery. The full-thickness rotator cuff tears of supraspinatus and infraspinatus will be classified into 2 categories based on longest dimension: SMALL = 0–1 cm; MODERATE = 1–3 cm. Definitive measurement of tear size will be made in surgery and used as a covariate in analysis (JOINTS Canada measurement protocol will be used).

#### Pre-operative exclusion criteria

1) Evidence of major joint trauma, infection, avascular necrosis, chronic dislocation, inflammatory or degenerative glenohumeral arthropathy, frozen shoulder, or previous surgery of the affected shoulder,

2) Evidence of significant cuff arthropathy with superior humeral translation and acromial erosion diagnosed by x-ray or other investigations,

3) Major medical illness (life expectancy less then 2 years or unacceptably high operative risk),

4) Unable to speak or read English,

5) Psychiatric illness that precludes informed consent,

6) Unwilling to be followed for 2 years.

#### Intra-operative exclusion criteria

7) Large, massive, or irreparable cuff tears extending into the subscapularis or teres minor which cannot be mobilized to the articular margin or repaired using one or both of the techniques (all-arthroscopic or mini-open),

8) Teres minor or subscapularis tears,

9) Inelastic and immobile tendon which cannot be advanced to articular margin,

10) Co-existing labral pathologies requiring repair with sutures (SLAP II-IV), Bankart lesions requiring repair, partial tears of biceps (more than 60% of thickness) requiring tenodesis or release.

### Sample size

We have previously completed a non-randomized pilot study (n = 36, JCM) comparing mini-open to all-arthroscopic rotator cuff repair and have data representative of the population we wish to sample for this study. We piloted a number of study outcomes, but our calculations are based on our planned primary outcome measure (WORC). From our pilot data, the baseline WORC scores were 1345/2100 (SD = 324) for mini-open patients and 1463(287) for arthroscopic repair. At 1 year, patients in the mini-open group had a mean score of 712.5 (SD = 650.7) and the patients in the all-arthroscopic group had a mean score of 940.6 (a difference of 228 points on the WORC). In our estimates of sample size requirements, we have used an estimated standard deviation of 450 since we suspect that the standard deviation observed in the pilot study at 1 year is larger than that expected at 2 years since patients are expected to be more stable in their outcomes by this time point. Further evidence to support our hypothesis comes directly from our pilot data where the estimate of standard deviations from the baseline assessment was 287 and 325. We will perform an analysis of covariance (ANCOVA) using the baseline WORC scores as a covariate to determine whether there is a statistically significant and clinically important difference between treatment groups at 2 years post-operative. We defined a clinically important difference as a 20% difference in the mean score between groups [47]. We used the following equation to calculate the sample size:

Calculation: N per group = 2((Z_α _+ Z_β_)^2^σ^2^(1-r^2^))/(δ)^2^, Where:

α = the probability of making a Type I error = 0.05

1-β = the power to detect a difference if one truly exists = 0.80, thus, β = 0.20

δ = 20% of the baseline mean score = 142.5*

r = correlation between baseline and post-operative data = 0.55

σ = standard deviation = 450

N per group = 2((1.96 + 0.84)^2 ^(450)^2^(1-0.55^2^))/(142.5)^2 ^= 109 patients per group

Next, we inflated this value by 15% to compensate for patients who will withdraw, who become lost to follow-up, or become procedural crossovers. Thus, the total sample size is 250 patients. If the estimate of standard deviation from our pilot work of 650 is accurate, our study will have 99% power to detect a difference between groups of the magnitude that was observed in our pilot work. This sample size will be also be adequate to detect moderate effects in secondary outcome measures, including self-report, impairment measures, and survey results. It is unlikely that this sample will be sufficient to detect difference in crossover rates as we expect the proportions will be very low. Nevertheless, we feel this is an important outcome to report so that future systematic reviews addressing expertise-based versus traditional randomization can address this issue.

### Arrangements for allocating participants to trial groups

Seven centres across Canada are participating in this trial (London, Calgary, Toronto, Vancouver, Edmonton, New Westminster, Winnipeg). All surgeons (n = 15) have been assigned to a design that will minimize the impact of expertise on study outcomes. Those with > 2 years recent experience in both procedures will require conventional design. Surgeons with primary expertise in only one procedure are paired with those with expertise in the alternative procedure from the same site and subject to expertise-based design. In both designs, the randomization scheme will be stratified by surgeon and compensation status (Workplace Safety and Insurance Board (WSIB), litigation or disability). We will use variable permuted block sizes to randomize patients on a 1:1 ratio; block sizes will remain confidential to the biostatistician to protect the integrity of the randomization scheme. Local research assistants will identify all patients (eligible participants, eligible non-participants and non-eligible), reasons for ineligibility or refusal, and obtain consent from eligible patients.

#### Conventional RCT

Patients will arrive at their surgical consultation where the surgeon will assess patient eligibility during history and physical examination, taking into account diagnostic information provided from pre-consultation MRI. The surgeon will explain the clinical trial and that by agreeing to participate, the patient has a 50:50 chance of receiving either method (mini-open or all-arthroscopic) to repair the torn rotator cuff tendon. The research assistant will obtain informed consent and contact the central randomization computer to obtain patient identification number and treatment assignment.

#### Expertise-based RCT

Patients will arrive at their surgical consultation where the surgeon will assess patient eligibility as per above. If eligible, the surgeon will explain the clinical trial and that by agreeing to participate, the patient has a 50:50 chance of receiving either method (mini-open or all-arthroscopic) to repair the torn rotator cuff tendon and that another surgeon from the same centre may perform the operation depending on group allocation; the surgeon performing the procedure will have expertise in performing that procedure. The research assistant will obtain informed consent and contact the central randomization computer to obtain patient identification number and surgeon (treatment) assignment. Two of the larger centres (Calgary and London) have some surgeons who qualify for the conventional RCT and others (pairs) for the expertise-based RCT so this factor will also be block randomized at these sites.

### Outcome measures

#### Primary outcome

##### Western Ontario Rotator Cuff Index (WORC)

The WORC was developed and validated as a self-report evaluative measure of quality of life specific to patients with rotator cuff disease [48]. The questionnaire consists of 21 items that are organized into five domains including pain and physical symptoms, sports and recreation, work, lifestyle, and emotional well-being. Reproducibility of the WORC has been demonstrated at 2 weeks and 3 months (0.96 and 0.93 respectively) [48]. In addition, this instrument has demonstrated ability to detect change. While studies vary, our recent work suggests that the WORC is more responsive than other scales [49]. All studies agree that the WORC is a valid measure of quality of life in patients experiencing rotator cuff problems when compared to alternative instruments [48,50]. All self-report measures will be administered by a blinded research assistant at each assessment point.

### Secondary outcomes

#### Complications/Cuff integrity

A standardized complications checklist has been developed. This will be used to monitor infection rate and other known post-surgical problems. MRI will be used to detect repair failure at 1 year post-operatively. Examination of the rotator cuff using MRI is highly correlated to surgical findings for full thickness tears, having a sensitivity and specificity of 85% and 83% for first-time shoulder surgery, and 84% and 91% for failed repairs [45]. For the purposes of this study, all MRI series will be reviewed by radiologists with special expertise in musculoskeletal imaging. The radiologists will conduct all measurements from the MRI images and will remain blinded to group allocation. Patients for whom MRI is contraindicated will be examined by ultrasound. Patients will undergo an MRI of the rotator cuff at 12 months post-operatively to evaluate cuff integrity. This is an appropriate time to ascertain whether the repair remains intact, as further biological recovery is unlikely. Cuff failure at this point may require revision surgery. Furthermore, delay of MRI would risk losing this key information if patients are lost to follow-up between the 1 and 2-year time points. Specifically, the MRI will document the size of the defect in the proximal to distal, humeral to bursal, superior to inferior direction in all four tendons, the muscle atrophy using the tangent sign (supraspinatus only), and the degree of fatty infiltration (supraspinatus and infraspinatus) as defined in previous work [7]. The appearance of the biceps tendon and any tear will be described (normal, tendinosis, partial-thickness tear (thinned or longitudinal split), or complete tear) and measured. Changes in the size of tear, muscle atrophy, and degree of fatty infiltration from the pre-operative state will be determined. This procedure has been used in an ongoing trial based at McMaster University and data collection will follow the same standardized format.

#### Self-report

The American Shoulder and Elbow Surgeons shoulder scale (ASES) [51] will provide a secondary measure of shoulder pain and disability; the Work Limitations Questionnaire [41] will measure work difficulties; and the SF-12 [42,52,53] will measure general health status. The ASES was selected as it is used internationally in shoulder studies and will allow comparability of data across trials. It has a single pain item and a 10-item function subscale. The Work Limitations Questionnaire [41] has been used to study loss of occupational productivity in other musculoskeletal disorders [41,54] and we have recently validated it for use in rotator cuff disorders (JCM manuscript in progress). The SF-12 is an abbreviated multi-item general health scale that evaluates the two domains of physical and mental health through summary scores. The SF-12 (version 2) correlates highly with the SF-36 [52,55,56] and has proved valid in a wide variety of populations and contexts. It was selected for this study because it allows a lesser respondent burden which was important given the burden of multiple self-report scales.

#### Physical impairments

##### ROM

A blinded research assistant will assess active and passive measurements of range of motion (ROM), including forward elevation, abduction, and internal and external rotation. These will be measured at all post-operative assessments using a Universal standard goniometer, which is reliable (ICC (range) = 0.85–0.99) for the measurement of shoulder ROM [57,58].

##### Strength

Strength of shoulder flexion, abduction, and internal and external rotation will be measured using a hand-held dynamometer. Patients will be sitting and have the arm placed in a neutral position. Maximum isometric strength will be measured over 3 trials using procedures which are known to produce reliable scores [59-62]. Each of the test sites will be provided a new dynamometer that has been calibrated to ensure validity of obtained measurements.

##### Composite Impairment Score (Constant)

The Constant is a composite summary score which addresses pain, activities of daily living, range of motion, and strength [63-69]. It takes approximately 10 minutes to administer [63]. This is not a self-administered questionnaire; the research assistant must work through each section with the patient [70]. For the purposes of this trial, a differential rate between shoulders will be calculated as it has been shown to provide a more precise estimate of function. In addition, we will use strength measures from dynamometry to provide more accurate estimates of muscular capability than possible with subjective ratings [63].

### Trial measures

We will monitor all aspects of recruitment and protocol compliance between study designs as study outcomes. In addition, we will measure patient and surgeon satisfaction with the study process and patients' proclivity for future trial participation.

#### Proposed methods for protecting against other sources of bias

##### Control over eligibility violations is under review of the central adjudication committee

The Central Adjudication Committee (CAC), blinded to patient group allocation, will review all operative reports and eligibility forms from all randomized patients to ensure they met eligibility criteria. This committee will consist of the principal investigator, an orthopaedic surgeon, and an epidemiologist. Any patient judged to be ineligible by the CAC, according to information available at the time of randomization, will be omitted from the final analysis and the reason for exclusion recorded. This will be recorded as a minor protocol violation.

##### Control of biases through blinding

Surgeons cannot be blinded. However, the patient, the data collector, radiologist, and data analyst are blinded to group allocation for the duration of the study and data analysis. As the primary outcome is self-report, bias is minimized.

##### Control of contamination and co-intervention

Patient intervention or crossovers within the expertise-based RCT are highly improbable. Crossovers from the conventional RCT may occur if surgeons feel a particular patient requires a specific type of surgery and, thus, are much more likely within this design. We will examine the records of all patients once surgery is completed to determine whether they received the assigned treatment. Crossovers will be recorded as major protocol violations. Surgical co-intervention is unlikely to occur at the time of surgery since we have specified in the eligibility criteria the concurrent procedures permitted. The biggest risk of co-intervention is if rehabilitation differs between the two groups. To avoid this, we have instituted a standardized rehabilitation protocol and will monitor adherence to this and the surgical protocol.

##### Ensuring reproducibility and reliability of measurements of outcome

The reliability and validity of the primary outcome measures has been established in previous studies [48,50,71]. JOINTS Canada currently uses the WORC as a primary outcome measure in other studies involving patients with rotator cuff disease. Thus, research assistants are familiar with its administration. Study centres are also familiar with methods to minimize nonresponse and to ensure compliance with completing forms. Experienced research assistants at each of the participating sites will perform range of motion and strength measurements. Standardized methods of measuring range of motion [58,72-74], strength [15,75-78], and tear size will be provided in the study manual and are similar to techniques previously used in other JOINTS Canada studies. All participating JOINTS Canada investigators have agreed upon these standardized methods. All research assistants have been trained to use these methods. Secondary outcome measures are also standardized scales that are reliable and valid including: the ASES shoulder scale [51,79-81], the SF-12, and the Work Limitations Questionnaire (WL-26) [41].

##### Control of biases relating to follow-up

The importance of complete follow-up to provide a valid estimate of outcomes in patients after rotator cuff surgery has been demonstrated [82]. We will use the following measures to ensure minimal loss to follow-up: 1) enrollment procedures will include the importance of compliance with follow-up and we will exclude patients who refuse 2-year follow-up; 2) study patients will supply, along with their own complete address and telephone information, similar contact information for 2 close associates; and 3) contact information will be updated at each study visit and by phone at 18 months. In addition, reminders of missed follow-up appointments are faxed to the research assistant who will immediately contact the patient to reschedule a missed appointment. Patients will be reimbursed for parking expenses associated with study. Patients who persist in noncompliance will be contact though telephone or internet survey for self-report outcome measures

### Planned analyses

Although considerable previous observational work suggest that both procedures are safe [21,26,28,30,32,83-86], safety will be evaluated by an external committee (DSMB) in conjunction with the steering committee (who will retain executive power). DSMB meetings will be held after the 1^st ^and 2^nd ^years of enrollment. The DSMB report will be evaluated by the steering committee and JOINTS Canada. No interim analyses are planned as both short and long-term outcomes are of interest, the probability of safety issues is very low, and because clinically important milestones exist across the spectrum of evaluations. As the primary outcome is quality of life, it is important to have a fully powered 2-year outcome.

We will conduct the analysis by pooling the data across designs (expertise-based and conventional), comparing the outcomes of patients who underwent mini-open versus all-arthroscopic. Pooling this data assumes that the design has no affect on the treatment effect. To test this assumption, we will test for heterogeneity of the results between studies using a formal assessment of the variability of results across studies by conducting statistical tests that provide an I^2 ^value. The I^2 ^value estimates the percentage of total variation in results across studies that are due to heterogeneity between studies rather than that due to chance. Low, moderate, and high levels of heterogeneity are roughly categorized by I^2 ^values of 25%, 50%, and 75% respectively. If the I^2 ^value is greater than 25%, we will perform a formal test of the interaction term to determine whether there was a significant difference in the treatment effect between groups, depending on which study design was used [87]. As indicated above, we will also monitor aspects of trial conduct including the potential numbers of patients at test sites, the recruitment rates, and the rate of study protocol violations.

In our primary analysis we will use an analysis of covariance (ANCOVA), where the baseline WORC score is used as a covariate, to compare the quality of life (WORC score) at 2 years post-operatively[88-91]. As a secondary analysis, we will conduct the same statistical test but will include age and tear size as covariates with the baseline score. All outcome measures with a continuous metric will be analyzed in the same manner (ASES, range of motion, MRI (degree of muscle atrophy, degree of fatty infiltration, remaining tear size), Work Limitations, strength, and SF-12). We will also conduct a repeated-measures ANCOVA to determine whether quality of life (as the primary outcome) changed differently over time between groups.

## Discussion

Surgical trials are urgently needed to define ideal surgical practices. Systematic reviews have been thwarted by the lack of such trials [38,39]. We found no previous trials on this question: Does all-arthroscopic or mini-open repair provide better outcomes, specifically quality of life, for patients with small or moderate-sized rotator cuff tears?

## Competing interests

The author(s) declare that they have no competing interests.

## Authors' contributions

RH and HR formed the original study team that developed the research question, wrote the pilot study protocol, obtained local ethics approval, obtained grant funding for a pilot study, and piloted the original study. RH proposed the study to JOINTS Canada. Joint Orthopaedic Initiative for National Trials of the Shoulder – Canada (JOINTS Canada) whose mandate is: "*An organization dedicated to raising the standards of education, assessment and care through multicentre clinical trials and to improve the quality of life for patients with shoulder disorders." *JOINTS Canada is an assembly of orthopaedic shoulder surgeons, epidemiologists, research coordinators, nurses, physiotherapists, and kinesiologists who have a common interest in shoulder research and who work collaboratively to conduct national trials in shoulder surgery. JOINTS Canada collectively prioritized this study, approved it, and contributed to protocol development. JCM wrote the study methods protocol for the multi-centre trial, wrote the grant application, and drafted this article. DB assisted with revisions to the study protocol and methods. All JOINTS Canada full members read and approved the final study protocol. All JOINTS Canada full members read and approved this manuscript. JOINTS Canada full members include: John Antoniou, Robert Balyk, Trevor Birmingham, Richard Boorman, Erin Boynton, Dianne Bryant, Darren Drosdowech, Jamie Dubberly, Ken Faber, Steven Gallay, Robert Hawkins, Laurie Hiemstra, Robert Hollinshead, Richard Holtby, Jordan Leith, Robert Litchfield, Ian Lo, Joe Lobo, Joy MacDermid, Peter MacDonald, Scott Mandel, Robert McCormack, Michael McKee, Nicholas Mohtadi, Jaydeep Moro, Helen Razmjou, Eric Renaud, and Danny Whelan. The JOINT members who are contributing patients to the trial are: Robert Balyk, Richard Boorman, Darren Drosdowech, Ken Faber, Steven Gallay, Robert Hollinshead, Richard Holtby, Jordan Leith, Ian Lo, Joe Lobo, Peter MacDonald, Robert McCormack, and Nicholas Mohtadi.

## Pre-publication history

The pre-publication history for this paper can be accessed here:


